# Physical activity, sedentary behaviour and smoking status among psychiatric patients in Singapore – a cross-sectional study

**DOI:** 10.1186/s12888-021-03103-7

**Published:** 2021-02-18

**Authors:** Vanessa Seet, Edimansyah Abdin, P. V. Asharani, Ying Ying Lee, Kumarasan Roystonn, Peizhi Wang, Fiona Devi, Laxman Cetty, Wen Lin Teh, Swapna Verma, Yee Ming Mok, Mythily Subramaniam

**Affiliations:** 1 Research Division, Institute of Mental Health, Singapore, Singapore; 2 Early Psychosis Intervention Programme, Institute of Mental health, Singapore, Singapore; 3 Department of Mood and Anxiety, Institute of Mental Health, Singapore, Singapore

**Keywords:** Physical activity, Sedentary behaviour, Smoking, Psychiatric patients

## Abstract

**Background:**

Unhealthy behaviours such as physical inactivity, sedentary behaviour and smoking have been found to be more prevalent in people with psychiatric disorders than in the general population, leading to increased mortality risk. The present study seeks to identify correlates of physical activity and sedentary behaviour among psychiatric patients in Singapore, as well as investigate differences in their physical activity patterns by smoking status.

**Methods:**

Participants (*n* = 380) were recruited from a tertiary psychiatric hospital in Singapore as part of a study on the prevalence and correlates of smoking among psychiatric patients. Physical activity levels and sedentary behaviour were measured using the Global Physical Activity Questionnaire (GPAQ) and analysed based on GPAQ guidelines. Chi-square analyses were conducted to examine differences in physical activity by smoking status, and logistic regression analyses to yield sociodemographic correlates of meeting physical activity guidelines (as recommended by the World Health Organization) and sedentary behaviour.

**Results:**

Education was found to be significantly associated with meeting recommended physical activity levels, while age and marital status were significantly associated with excessive sedentary behaviour. Additionally, while no significant differences were found among current, former and non-smokers across all types of physical activity engagement levels, there was a high prevalence of inadequate physical activity (43.2%) and excessive sedentary behaviour (38.8%) among participants.

**Conclusion:**

Given the high prevalence of inadequate physical activity and excessive sedentary behaviour among current, former and non-smokers with psychiatric disorders, programmes aimed at increasing physical activity and lowering sedentary behaviour levels should be integrated into targeted treatment plans to improve clinical outcomes.

## Background

Physical inactivity and smoking have been extensively established as leading risk factors for non-communicable diseases (NCDs) [1, 2]. Physical inactivity, the fourth leading cause for premature mortality, accounts for approximately 1.6 million of the deaths from NCDs annually [3] and poses a great economic burden worldwide [4]. Despite regional implementation plans by countries to boost physical activity in response to the World Health Organization (WHO) Global Action Plan on NCDs, these plans have not resulted in any significant increase in physical activity levels across the population [5]. With regards to smoking, the growth of the global population over the decades has contributed to an increase in the absolute number of smokers by almost 280 million from 1980 to 2012 [6]. Currently, the use of tobacco accounts for more than 7.2 million of the 41 million deaths attributable to NCDs each year, and this number is only set to increase over the years as a result of the increase in number of smokers, thereby cementing the status of smoking as a major contributor of mortality rates [3]. Thus, the figures and health ramifications pertaining to physical inactivity and smoking are serious enough to warrant active measures for controlling people’s engagement in these two unhealthy behaviours [1, 2].

In the general population, the global prevalence of physical inactivity is 27.5%, ranging from 16.3 to 39.1% across different regions [7]. However, this figure is higher among those with psychiatric disorders [8]. Compared to people without a depressive disorder, people with major depressive disorder were found to have significantly lower levels of engagement in physical activity, greater levels of sedentary behaviour, and a lower likelihood of meeting WHO-recommended physical activity guidelines [9]. Among people with schizophrenia, Stubbs and colleagues found lower levels of engagement in moderate and vigorous physical activity when compared to people without [10], while Soundy and colleagues found in their meta-analysis significantly greater levels of sedentary behaviour among people with schizophrenia, as opposed to healthy controls matched for age and gender [11]. In a similar vein, the current prevalence for smoking in the general population varies across countries and regions, ranging from about 24 to 48% in men, and from about 2 to 22% in women [12]. Again, smoking is consistently more pervasive among people with psychiatric disorders, increasing their susceptibility to the debilitating health risks of smoking. In a study by Lawrence, Mitrou and Zubrick, it was found that in the United States and in Australia, the prevalence of smoking in adults who met the criteria for a psychiatric disorder was approximately twice that of adults who did not [13], a finding previously demonstrated by Lasser, Boyd and Woolhandler [14].

With the disproportionately high prevalence of smokers and the physically inactive among people with psychiatric disorders, and in turn the higher mortality rates these people face from the complications raised by these two behaviours [8, 15], it is therefore pertinent to address this problem in this specific group. Interestingly, in addition to general physical health benefits, reduction of the risk of premature all-cause mortality, and primary prevention of NCDs [16–18], physical activity may also help to reduce cigarette cravings as well as tobacco withdrawal symptoms, in turn improving smoking behaviour [19, 20]. Moreover, physical activity has been found to alleviate depressive symptoms in people with major depressive disorder, as well as positive and negative symptoms in people with schizophrenia-related disorders [21].

Given the modifiability of physical inactivity and smoking, and the importance of physical activity in reducing health complications, a better understanding of physical activity and sedentary behaviour correlates, as well as smoking behaviours among people with psychiatric disorders is essential for better, more targeted interventions in promoting healthy behaviours and in turn improving their general health. While the association between smoking and physical activity has been demonstrated in the general population [22–25], the same cannot be said for the psychiatric population, with studies usually investigating the two factors separately [8, 13, 15]. Hence, this paper aims to bridge the research gap in this area by examining associations between physical activity, sedentary behaviour and smoking among people with psychiatric disorders. Specifically, we investigate factors influencing sufficient physical activity, defined as meeting WHO-recommended physical activity levels for health maintenance, and sedentary behaviour, as well as differences in physical activity levels by smoking status among people with depressive disorders and schizophrenia spectrum and other psychotic disorders.

## Methods

### Participants

The data used in this analysis was collected as part of a study on the prevalence and correlates of smoking and cessation among patients with depressive disorders or schizophrenia and other psychotic disorders in a tertiary psychiatric hospital in Singapore [26]. Participants were recruited from the Institute of Mental Health, Singapore over a one-year period. Potential participants were approached in the outpatient clinic by the study team or referred by clinicians, and were recruited for the study if they met the following criteria: being 21 to 65 years of age, having a primary diagnosis of either a depressive disorder (for example, major depressive disorder or dysthymia) or schizophrenia spectrum and other psychotic disorders (for example, schizophrenia or psychotic disorder), and being cognitively able to give informed consent. Participants were excluded from the study if they were below 21 years of age, not a registered patient of IMH (ie were seeking psychiatric treatment from other clinics or hospitals at point of contact), had other primary psychiatric disorders, or if they did not have the cognitive capacity to consent. Information on participants’ clinical diagnoses, which were based on criteria from the fourth edition of the Diagnostic and Statistical Manual of Mental Disorders (DSM-lV), were obtained from their electronic medical records. Participants underwent an interviewer-administered survey after giving their written informed consent to take part in the study, and all participants were clinically stable at the point of the interview. Ethics approval was obtained from the Institutional Research Review Board and the National Healthcare Group Domain Specific Review Board (Ref: 2018/00772), and all study procedures were carried out in accordance with the relevant guidelines and regulations.

### Global physical activity questionnaire (GPAQ)

The GPAQ is an interviewer-administered questionnaire that was developed by WHO as a physical activity surveillance tool across populations, and as part of their STEPwise approach to surveillance of NCDs and associated risk factors [27, 28]. The GPAQ has demonstrated reasonable validity and reliability in studies across multiple populations [29, 30]. In this questionnaire, physical activity was categorised into three main domains – work or training, travelling (via walking and/or cycling), and leisure activities. This was further divided into vigorous- or moderate-intensity work activity, and vigorous- or moderate-intensity leisure activity. Participants were asked if they engaged in such activities for at least 10 min at a time in a typical week, as well as the number of days and amount of time they spent per day doing such activities. Definitions and examples of such activities were also given to participants to aid in their recall and answers. Lastly, participants also answered a question on sedentary behaviour, pertaining specifically to the amount of time they spent sitting or reclining on a typical day. GPAQ data was processed and analysed using the analysis guide provided by WHO [31]. For activity level, high activity level was computed as either a minimum of 3 days of vigorous-intensity activities and 1500 metabolic equivalent (MET) minutes in a week, or at least 7 days of walking, moderate- or vigorous-intensity activities (MVPA) and 3000 MET minutes per week. Moderate activity level was computed as not meeting the criteria for high levels, but either having at least 3 days of vigorous-intensity activity for 20 min or more per day, or at least 5 days of moderate-intensity activity or walking for 30 min or more per day, or at least 5 days of combined walking and MVPA with a minimum of 600 MET-minutes per week. Low activity level was computed as not meeting the criteria for either high or moderate physical activity level.

Criteria for meeting WHO-recommended physical activity guidelines for health maintenance were also computed based on the recommended cut-off values from the GPAQ analysis guide, which was either two and a half hours of moderate-intensity activity, 1 h and 15 min of vigorous-intensity activity, or at least 600 MET-minutes of an equivalent combination of moderate- and vigorous-intensity activity, within a week [31].

### Sociodemographic and health-related information

Participants were asked about their sociodemographic information including age, gender, ethnicity, education level, marital status, and employment status prior to completion of the GPAQ. They were also asked if they were currently smoking regularly (current smokers), used to smoke but have since quit (former smokers), or had never picked up smoking (non-smokers). Additionally, participants’ body-mass indexes (BMIs) were calculated based on their height and weight which were measured after the survey, and categorised according to WHO international cut-offs where below 18.5 kg/m^2^ was categorised as underweight, 18.5 to 24.9 kg/m^2^ normal weight, 25 to 29.9 kg/m^2^ overweight, and 30 kg/m^2^ and above obese [32].

### Statistical analyses

Applying the cut-offs from the GPAQ guide, physical activity was categorised into three levels – high, moderate, and low [31]. Physical activity was also used to assess whether participants’ activity engagement met WHO-recommended levels for health maintenance, for which binary variables (yes/no) were created. Sedentary behaviour duration was categorised into two levels – 7 h and below, and more than 7 h. This cut-off was derived from Ku and colleagues’ meta-regression analyses, which demonstrated that all-cause mortality risks increased significantly at more than 7 h of sedentary behaviour per day for self-reported measurement [33]. Descriptive characteristics for sociodemographic variables and engagement in physical activity domains are presented as cell counts and percentages. Cross-tabulations and chi-square tests were applied to examine differences in physical activity level among participants by gender, ethnicity, clinician’s diagnosis, and marital, employment and current smoking status, as well as differences in engagement in the different physical activity domains by smoking status. Associations between physical activity level and age, education level and BMI were examined using Kendall’s tau-c. Finally, forced entry binary logistic regression analyses were conducted to investigate associations between sociodemographic variables, and WHO-recommended physical activity engagement and sedentary behaviour respectively, and the resulting odds ratios (ORs) and 95% confidence intervals (CIs) reported. All statistical analyses were conducted with IBM SPSS Statistics for Windows, version 25.0 [34], and statistical significance was indicated by a cut-off value of *p* < 0.05.

## Results

### Social demographics and physical activity prevalence

Participants’ social demographic characteristics are displayed in Table 1, stratified by physical activity level. In total, 380 people participated in the study; people of other ethnicities were excluded from analysis on account of their small number and heterogeneity, leaving 373 participants’ data to be analysed. The results of chi-square analyses showed significant differences in physical activity level by marital status only, χ^2^ (4, *N* = 373) = 14.9, *p* < .05. Bonferroni-corrected post hoc comparisons of physical activity level by marital status revealed that significantly fewer married participants engaged in high levels of physical activity than in low and moderate levels, while participants who were single engaged in high physical activity levels significantly more than the other two levels. Overall, 36.7% of all participants engaged in low levels of physical activity per week, 32.4% engaged in moderate levels, and 30.8% engaged in high levels of physical activity.

**Table 1:** Sociodemographic characteristics of psychiatric patients by physical activity level

	Total		Low physical activity		Moderate physical activity		High physical activity				
	n	%	n	%	n	%	n	%	χ^2^	*τ*^*c*^	*p*
Age									–	0.09	0.101
21–40	183	49.1	59	32.2	63	34.4	61	33.3			
41–65	190	50.9	78	41.1	58	30.5	54	28.4			
Gender									4.58	–	0.101
Male	207	55.5	76	36.7	59	28.5	72	34.8			
Female	166	44.5	61	36.7	62	37.3	43	25.9			
Ethnicity									6.12	–	0.191
Chinese	279	74.8	108	38.7	94	33.7	77	27.6			
Malay	55	14.7	18	32.7	14	25.5	23	41.8			
Indian	39	10.5	11	28.2	13	33.3	15	38.5			
Educational level									–	0.04	0.396
Primary school	50	13.4	26	52.0	13	26.0	11	22.0			
Secondary school	100	26.8	33	33.0	29	29.0	38	38.0			
Pre-university (JC/diploma/ITE)	162	43.4	58	35.8	57	35.2	47	29.0			
University	61	16.4	20	32.8	22	36.1	19	31.1			
Marital status									14.9	–	0.005
Married	62	16.6	24	38.7	30	48.4	8	12.9			
Single	259	69.4	91	35.1	76	29.3	92	35.5			
Separated/divorced/ widowed	52	13.9	22	42.3	15	28.8	15	28.8			
Employment status									5.16	–	0.271
Employed	171	45.8	56	32.7	56	32.7	59	34.5			
Unemployed	167	44.8	70	41.9	50	29.9	47	28.1			
Economically inactive	35	9.4	11	31.4	15	42.9	9	25.7			
BMI									–	0.03	0.567
Underweight (below 18.5)	11	3.0	4	36.4	6	54.5	1	9.1			
Normal weight (18.5 - 24.9)	128	34.6	50	39.1	40	31.3	38	29.7			
Overweight (25–29.9)	128	34.6	43	33.6	42	32.8	43	33.6			
Obese (30 and above)	103	27.8	39	37.9	32	31.1	32	31.1			
Current smoking status									9.02	–	0.061
Non-smoker	194	52.0	70	36.1	73	37.6	51	26.3			
Past smoker	34	9.1	12	35.3	13	38.2	9	26.5			
Current smoker	145	38.9	55	37.9	35	24.1	55	37.9			
Clinician’s diagnosis									1.37	–	0.505
Depressive disorder	173	46.4	59	34.1	56	32.4	58	33.5			
Schizophrenia spectrum or other psychotic disorder	200	53.6	78	39.0	65	32.5	57	28.5			

Note: *JC* Junior College; *ITE* Institute of Technical Education; *BMI* Body mass index

The proportions of participants engaging in the different activity domains, stratified by current smoking status, are displayed in Table 2. The percentage of participants engaging in high levels of sedentary behaviour (over 7 h per day) was 38.8, and 43.2% of participants did not manage to meet the minimum required amount of physical activity as recommended by WHO. Among current smokers, former smokers and non-smokers, there was no significant difference in weekly engagement in work-related activity, travel activity, leisure activity, sedentary behaviour, or whether WHO-recommended physical activity guidelines were met.

**Table 2:** Physical activity engagement by smoking status

	Total		Current smokers		Former smokers		Non-smokers		
	n	%	n	%	n	%	n	%	*p*
Work-related MVPA									0.623
Yes	193	52	80	55.2	17	50	96	50	
No	178	48	65	44.8	17	50	96	50	
Travel activity									0.710
Yes	270	72.4	102	70.3	24	70.6	144	74.2	
No	103	27.6	43	29.7	10	29.4	50	25.8	
Leisure MVPA									0.347
Yes	193	51.7	70	48.3	21	61.8	102	52.6	
No	180	48.3	75	51.7	13	38.2	92	47.4	
Sedentary behaviour									0.207
< = 7 h of SB	227	61.2	90	62.5	16	47.1	121	62.7	
> 7 h of SB	144	38.8	54	37.5	18	52.9	72	37.3	
Meeting WHO PA recommendations									0.417
Yes	212	56.8	88	60.7	17	50	107	55.2	
No	161	43.2	57	39.3	17	50	87	44.8	

Note: *MVPA* Moderate-vigorous physical activity; *SB* Sedentary behaviour; *WHO* World Health Organization; *PA* Physical activity

### Correlates of meeting WHO-recommended physical activity guidelines and sedentary behaviour

The results of binary logistic regression on the odds of meeting WHO-recommended physical activity levels and excessive sedentary behaviour are presented in Table 3. Only education level was significantly associated with engaging in WHO-recommended levels of physical activity. Specifically, those whose highest qualification was secondary school had significantly higher odds of meeting WHO-recommended physical activity levels as opposed to those who only attained a primary education (OR = 2.40, 95% CI = 1.15–5.01, *p* = 0.02). Despite not reaching statistical significance, those whose education was above primary school had consistently higher odds of meeting the recommended physical activity levels across all educational levels.

**Table 3:** Sociodemographic correlates of engagement in WHO-recommended levels of physical activity and excessive sedentary behaviour

	Odds of meeting WHO-recommended PA levels					Odds of engaging in excessive SB (> 7 h)				
	OR	95% CI			*p*	OR	95% CI			*p*
Age										
41–65 (reference)										
21–40	1.15	0.69	–	1.92	0.584	2.35	1.36	–	4.07	0.002
Gender										
Female (reference)										
Male	1.11	0.68	–	1.81	0.680	1.02	0.61	–	1.69	0.942
Ethnicity										
Chinese (reference)										
Malay	1.55	0.80	–	3.01	0.194	1.25	0.65	–	2.43	0.503
Indian	1.36	0.64	–	2.87	0.424	1.04	0.48	–	2.24	0.920
Education level										
Primary school (reference)										
Secondary school	2.40	1.15	–	5.01	0.020	1.59	0.70	–	3.64	0.271
Pre-university (JC/diploma/ITE)	1.88	0.89	–	3.96	0.097	1.35	0.58	–	3.11	0.485
University	2.30	0.95	–	5.56	0.064	1.75	0.67	–	4.54	0.251
Marital status										
Married (reference)										
Single	1.53	0.82	–	2.84	0.178	1.89	0.92	–	3.86	0.081
Separated/divorced/widowed	1.45	0.65	–	3.25	0.361	2.62	1.07	–	6.37	0.034
Employment status										
Unemployed (reference)										
Employed	1.20	0.76	–	1.91	0.433	1.09	0.67	–	1.79	0.718
Economically inactive	1.10	0.51	–	2.39	0.806	1.41	0.62	–	3.19	0.408
Current smoking status										
Non-smoker (reference)										
Former Smoker	0.79	0.36	–	1.73	0.552	2.12	0.94	–	4.79	0.072
Smoker	1.29	0.75	–	2.22	0.355	1.10	0.62	–	1.93	0.748
BMI										
Normal weight (18.5–24.9) (reference)										
Underweight (below 18.5)	1.17	0.31	–	4.33	0.818	3.21	0.80	–	12.86	0.100
Overweight (25–29.9)	1.04	0.62	–	1.75	0.880	1.19	0.69	–	2.07	0.534
Obese (30 and above)	1.10	0.62	–	1.94	0.748	1.14	0.63	–	2.08	0.661
Clinical diagnosis										
Schizophrenia spectrum or other psychotic disorder (reference)										
Depressive disorder	1.20	0.74	–	1.95	0.460	1.54	0.93	–	2.55	0.096

Note: *JC* Junior College; *ITE* Institute of Technical Education; *BMI* Body mass index; *SB* Sedentary behaviour; *WHO* World Health Organization; *PA* Physical activity

With regards to excessive sedentary behaviour, those who were 21 to 40 years of age were more likely to engage in more than 7 h of sedentary activity per day versus those who were 41 to 65 years of age (OR = 2.35, 95% CI = 1.36–4.07, *p* = 0.002). Additionally, people who were separated, divorced or widowed had a higher likelihood of engaging in excessive sedentary behaviour when compared with their married counterparts (OR = 2.62, 95% CI = 1.07–6.37, *p* = 0.034).

## Discussion

Expanding on previous studies that investigated smoking and physical activity separately [9, 10, 14, 35], the present study examined differences in physical activity level by sociodemographic variables, as well as in physical activity engagement by smoking status among people with schizophrenia spectrum and other psychotic disorders and depressive disorders. In our chi square analyses, we found a significant difference in physical activity level by marital status – fewer married participants engaged in high levels of physical activity, while the opposite was seen for unmarried participants. This is line with Rapp and Schneider’s longitudinal study, which found that married men and women engaged in lower levels of physical activity than their single counterparts [36]. A possible explanation for this is the marriage market hypothesis, which postulates that people who are single tend to engage in behaviours that increase their social desirability in order to improve their marriage market prospects – such behaviours include turning to higher levels of physical activity to lower their BMI [37]. On the other hand, those who are married and thus not as concerned about maintaining their social desirability may find their time and effort being taken up by other priorities besides vigorous physical activity, choosing instead to spend time on moderate and less taxing physical activity [37].

With regards to smoking status and physical activity, contrary to the studies by Heydari and colleagues, and Bobes, Arango, Garcia-Garcia and Rejas**,** both of which found lower likelihoods of adequate physical activity among smokers than non-smokers [25, 38], we found no significant differences in physical activity engagement among smokers, former smokers and past smokers. This discrepancy might be due to the different conceptualisations of physical activity used in the studies – in their study involving patients with schizophrenia spectrum disorders, Bobes and colleagues defined physical activity as self-reports of habitual engagement in any exercise, including walking, at a minimum occurrence of twice per week [38]. In their population study with healthy adults in Tehran, Heydari and colleagues, on the other hand, computed physical activity using self-reported time spent on inactive (sleeping, lying down and sitting) and active (standing, walking and running) positions in a day, and then calculated adequacy of physical activity using the proportion of time spent on both types of positions in relation to each other [25]. Finally, our calculations on physical activity engagement were based on GPAQ analysis and WHO-recommended physical activity guidelines, and included moderate and vigorous activity spent at work, at leisure, and during travel as well.

Nevertheless, regardless of smoking status, approximately one-third of people with schizophrenia spectrum and other psychotic disorders or depressive disorders were found to engage in low levels of physical activity, and close to half did not engage in sufficient levels of physical activity as recommended by WHO. This result is in line with Vancampfort and colleagues’ meta-analysis, whose results showed that 44.9% of people with severe mental illness did not meet WHO-recommended physical activity levels [8]. Other studies have reported similar results on the high prevalence of insufficient physical activity in people with psychiatric disorders [39, 40]. Compared to Guthold, Stevens, Riley and Bull’s pooled analysis of global population surveys, where they found the prevalence of inadequate physical activity in Singapore’s general population to be 36.5% [7], and Subramaniam and colleagues’ study on physical activity among multimorbid patients, where they found insufficient activity among 53.8% of Singaporeans with multiple chronic conditions [41], the proportion of participants in the current study who are not active enough stands at 43.2%. This is a worrying trend among people with psychiatric disorders, for whom the health benefits of physical activity are substantial but not being tapped into. Hence, it may be prudent for mental healthcare providers to address this area with their patients and integrate physical activity into their treatment plan. One such activity that can be incorporated is walking, which has been found to have a lower entry point and is the preferred method of physical activity among people with psychiatric disorders [40, 42, 43]. Overall, the quality of mental healthcare for not just smokers, but also former and non-smokers with psychiatric disorders, can only be improved by the integration of physical activity into treatment plans, in turn yielding better general health outcomes.

Binary logistic regression analysis also revealed the association between education level and physical activity engagement. Compared to those who received only up to a primary school education, people whose educational qualifications were above the primary level had higher odds of meeting WHO guidelines for physical activity levels, but only secondary school attainment showed a statistically significant association. This is consistent with previous studies which have demonstrated the link between lower educational level and inadequate physical activity, in both the general and psychiatric populations [8, 44–46]. This may reflect a greater awareness among people with higher education of the health benefits that engaging in recommended levels of physical activity can bring about in terms of health promotion and disease prevention, and their ease of access to such activity [45, 47]. In addition, a higher education may be indicative of higher socio-economic status, which contributes to people’s economic ability to engage in physical activity, especially for leisure [44, 48].

With regards to sedentary behaviour, statistically significant associations were found for age and marital status. Those who were separated, divorced or widowed were more likely to engage in excessive sedentary behaviour compared to those who were married, lending credence to previous studies which found negative associations between sedentary behaviours and being married [49–51]. These findings suggest that marriage plays a protective role against sedentary behaviour, perhaps through spousal support in encouraging healthy behaviours such as physical activity [52]. For age, those who were 21 to 40 years of age had a higher likelihood of engaging in excessive sedentary behaviour, compared to their older counterparts who were 41 to 65 years of age. While this finding is in direct contrast with other studies which have consistently found age to be positively correlated with sedentary behaviour [53], a plausible explanation may be the preference that older people have for walking, for both leisure and travelling [43, 54], as opposed to young adults who are more inclined to non-active modes of travel such as public or car transport [55]. In addition, the increased importance older adults place on maintaining their general health with physical activity [56, 57], as opposed to younger adults, may also contribute to their lower levels of sedentary activity.

The limitations of this study should be considered when interpreting its findings. First, as the study utilised self-report measures, participants might have been susceptible to overestimation of physical activity and underestimation of sedentary behaviour [58, 59]. In order to minimise this bias, interviewers probed for specificity in the type of physical activity and amount of time participants spent engaging in the activity and provided relevant examples to aid in participants’ recall. Secondly, information on psychotropic medication and symptom severity, which have been shown to influence physical activity [8, 10], was not captured in this study. Finally, people with other disorders were not included in the study as the bulk of the clinical population in the Institute of Mental Health comprises patients with depressive disorders or psychosis-related disorders, and the study was thus focused on the outcomes and smoking-related characteristics of people with these disorders. Given the non-probability sampling design of the study and the limited types of disorders included in this study, as well as the lack of a control group of participants with no mental disorder, the results of the study may not be generalisable to this population at large. Future research could expand the scope of the current study by incorporating objective measurements of physical activity and sedentary behaviour over multiple timepoints while controlling for the effects of medication and symptom severity, as well as including people with other mental disorders such as anxiety and bipolar disorder, to portray a more complete picture of engagement patterns in these behaviours. Nevertheless, the study adds insight into physical activity and sedentary behaviour patterns among people in Singapore with depressive disorders, and schizophrenia spectrum and other psychotic disorders, and provides preliminary information that will aid in treatment planning and delivery for these groups of people.

## Conclusion

The present study sought to examine behavioural patterns in physical activity and sedentary behaviour among current, former and non-smokers with depressive disorders and schizophrenia spectrum and other psychotic disorders in a Singapore tertiary psychiatric institution. Inadequate physical activity, defined as not meeting WHO-recommended physical activity guidelines, was found to be common among patients regardless of smoking status. Analyses further revealed that physical activity was associated with education, while sedentary behaviour was associated with age and marital status. Despite its limitations, the findings from this study can be used to identify sociodemographic groups that are more prone to physical inactivity and sedentary behaviour, which can in turn facilitate treatment design for improved clinical outcomes in psychiatric patients.

## Data Availability

The datasets used and/or analysed during the current study are available from the corresponding author on reasonable request.

## Abbreviations

GPAQ: Global physical activity questionnaire
NCD: Non-communicable disease;
WHO: World health Organization
DSM-lV: Diagnostic and statistical manual of mental disorders, fourth edition
MET: Metabolic equivalent
MVPA: Moderate- or vigorous-intensity activities
BMI: Body mass index
OR: Odds ratio
CI: Confidence interval
